# Cytotoxicity and Microbiological Properties of Copolymers Comprising Quaternary Ammonium Urethane-Dimethacrylates with Bisphenol A Glycerolate Dimethacrylate and Triethylene Glycol Dimethacrylate

**DOI:** 10.3390/ma16103855

**Published:** 2023-05-20

**Authors:** Marta W. Chrószcz-Porębska, Izabela M. Barszczewska-Rybarek, Alicja Kazek-Kęsik, Izabella Ślęzak-Prochazka

**Affiliations:** 1 Department of Physical Chemistry and Technology of Polymers, Faculty of Chemistry, Silesian University of Technology, Strzody 9 Str., 44-100 Gliwice, Poland; marta.chroszcz@polsl.pl; 2 Department of Inorganic Chemistry, Analytical Chemistry and Electrochemistry, Faculty of Chemistry, Silesian University of Technology, Krzywoustego 6 Str., 44-100 Gliwice, Poland; alicja.kazek-kesik@polsl.pl; 3 Biotechnology Centre, Silesian University of Technology, Krzywoustego 8 Str., 44-100 Gliwice, Poland; izabella.slezak-prochazka@polsl.pl; 4 Department of Systems Biology and Engineering, Faculty of Automatic Control, Electronics and Computer Science, Silesian University of Technology, Akademicka 16 Str., 44-100 Gliwice, Poland

**Keywords:** quaternary ammonium compounds, composite dental resin, cytotoxicity test, antifungal agents, antibacterial agents

## Abstract

Using dental composite restorative materials with a copolymeric matrix chemically modified towards bioactive properties can help fight secondary caries. In this study, copolymers of 40 wt.% bisphenol A glycerolate dimethacrylate, 40 wt.% quaternary ammonium urethane-dimethacrylates (QAUDMA-m, where m represents 8, 10, 12, 14, 16 and 18 carbon atoms in the N-alkyl substituent), and 20 wt.% triethylene glycol dimethacrylate (BG:QAm:TEGs) were tested for (i) cytotoxicity on the L929 mouse fibroblast cell line; (ii) fungal adhesion, fungal growth inhibition zone, and fungicidal activity against *C. albicans*; and (iii) bactericidal activity against *S. aureus* and *E. coli*. BG:QAm:TEGs had no cytotoxic effects on L929 mouse fibroblasts because the reduction of cell viability was less than 30% compared to the control. BG:QAm:TEGs also showed antifungal activity. The number of fungal colonies on their surfaces depended on the water contact angle (WCA). The higher the WCA, the greater the scale of fungal adhesion. The fungal growth inhibition zone depended on the concentration of QA groups (*x_QA_*). The lower the *x_QA_*, the lower the inhibition zone. In addition, 25 mg/mL BG:QAm:TEGs suspensions in culture media showed fungicidal and bactericidal effects. In conclusion, BG:QAm:TEGs can be recognized as antimicrobial biomaterials with negligible biological patient risk.

## 1. Introduction

Materials with methacrylate matrices are the most commonly used in dentistry [1]. They have good utility properties, low cytotoxicity, and negligible microbial activity [2,3,4]. Although dentistry offers a wide range of excellent materials, dental caries are observed on a large scale [5,6]. A large part of this problem concerns children [7,8]. Materials with antimicrobial properties could help to fight this disease [9].

Methacrylate monomers with quaternary ammonium (QA) groups are suitable components that, when chemically incorporated into the structure of the organic matrix of dental composite restorative materials (DCRM), give them anti-microbiological activity [10,11,12]. This type of modification involves the free radical copolymerization of QA monomers with common dental dimethacrylates such as bisphenol A glycerolate dimethacrylate (Bis-GMA), urethane-dimethacrylate (UDMA), and triethylene glycol dimethacrylate (TEGDMA), which leads to the formation of polymer networks with high crosslink density [13,14]. 

Methacrylates with QA groups include monomethacrylates (mono-QAMs) [15,16,17,18,19,20,21] and dimethacrylates (di-QAMs) [22,23,24,25,26,27,28,29,30]. Mono-QAMs such as 12-methacryloyloxydodecylpyridinium bromide [15], and N,N-dimethylaminoethyl methacrylate (DMAEMA) derivatives with bromide [16,17], iodide [18,19,20], or chloride [21] counter ions were the first to be obtained. DCRMs modified with the above mono-QAMs showed high antibacterial activity against many strains of bacteria, such as *Streptococcus mutans*, *Actinomyces viscosus*, and *Lactobacillus casei* [15,16,17,19,20,21]. However, it turned out that they caused deterioration of the physical and mechanical properties of the DCRM matrix, mainly by increasing its elasticity, water absorption, and free monomer leachability [17,18,19,22]. 

In the hope of eliminating this flow, di-QAMs were developed. The presence of two methacrylate groups in the monomer molecule theoretically gives it a chance to build onto the structure of the polymer network at both ends [22]. Di-QAMs include monomers obtained via the reaction of DMAEMA with dibromo alkanes [23] and urethane-dimethacrylates obtained via the reaction of quaternized N-methyldiethanolamine with 2-(methacryloyloxy)ethyl isocyanate [24,25], or 2-hydroxyethyl methacrylate and 5-isocyanato-1-(isocyanatomethyl)-1,3,3-trimethylcyclohexane [26,27]. Due to the aliphatic structures of these di-QAMs, polymers with no satisfactory properties were obtained. Therefore, the quaternized Bis-GMA derivative was developed. It was obtained from quaternized bisphenol A glycerolate diethylamine and methacryloyl chloride. The polymers modified with quaternized Bis-GMA derivative showed increased values of selected physical properties (water sorption and solubility) [28]. However, high antibacterial activity was the standard and essential feature of dimethacrylate copolymers containing di-QAMs and related materials. It was observed against the following bacteria strains: *S. mutans*, *Escherichia coli*, *Streptococcus aureus*, *Pseudomonas aeruginosa*, and *Bacillus subtillis* [23,24,25,26,27,28]. 

On the other hand, published literature shows that QA monomers may increase the cytotoxicity of methacrylate copolymers. Li et al. [30] reported that the addition of 10 wt.% DMAEMA dimethacrylate derivative with one QA group did not change the Bis-GMA:HEMA cytotoxicity determined on a human gingival fibroblast cell line. Antonucci et al. [29] used the same monomer to test its influence on the cytotoxicity of Bis-GMA:TEGDMA. Any difference in cell viability (RAW 264.7 macrophage-like cell line) was observed between the copolymer modified with 10 wt. % QA monomer and the reference. When the QA monomer concentration increased to 20 and 30 wt.%, reductions of no more than 30% were observed in cell viability. It was also found that monomers with two QA groups increased copolymer cytotoxicity at a lower concentration. Manouchehri et al. [23] reported that 1 wt.% DMAEMA dimethacrylate derivatives with two QA groups caused a one-quarter decrease in cell viability (human foreskin fibroblast) to the reference. Makvandi et al. [28] tested the cytotoxicity of Bis-GMA:TEGDMA modified with 5 to 15 wt.% Bis-GMA derivative containing two QA groups. They observed that the introduction of QA monomer caused a decrease in cell viability (L929 mouse fibroblast) from 10 to 40%. 

The bioactivity of compounds containing QA groups, including dimethacrylates, encouraged further research towards developing novel monomer chemical structures and their compositions that result in polymeric materials combining high antibacterial activity, low cytotoxicity, and appropriate utility properties. 

**Figure 1:**
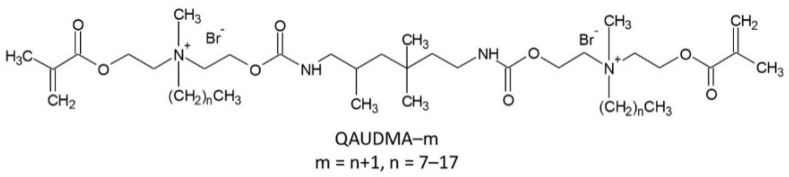
Chemical structure of QAUDMA-m monomers.

Our research group has been working on new di-QAMs and their copolymers. These monomers include a series of urethane-dimethacrylates with two quaternary ammonium groups (QAUDMA-m, m being related to the length of the N-alkyl substituent and corresponding to 8, 10, 12, 14, 16, and 18 carbon atoms) (Figure 1) [31]. QAUDMA-m monomers are similar in chemical structure to the so-called urethane dimethacrylate (UDMA) monomer due to the following structural elements: (i) the 1,6-diisocyanato-2,4,4-trimethylhexylene (TMDI) core, (ii) two arms attached to the core by carbamate linkages, and (iii) methacrylate group at the end of each arm. The presence of the QA group in each arm distinguishes QAUDMA-m from UDMA.

The results of the initial study showed that the QAUDMA-m monomers were highly viscous resins, had appropriate transparency, polymerized to high degrees of conversion (*DC*), and were characterized by low polymerization shrinkage (*S_e_*) [31]. These promising characteristics of QAUDMA-m monomers qualified them for further research to assess their influence on the properties of copolymers with common dental dimethacrylates.

Copolymers of 60 wt.% QAUDMA-m with 40 wt.% TEGDMA (QAm:TEGs) were first investigated by analogy to the 60 wt.% Bis-GMA with 40 wt.% TEGDMA copolymer (BG:TEG), representing standard DCRM matrix [32,33]. QAm:TEGs revealed significantly higher antibacterial activity against *S. aureus* and *E. coli* than BG:TEG, which depended on the number of carbon atoms in the N-alkyl substituent (*Cm*). In the case of *S. aureus*, QA14:TEG, on whose surface no bacteria colonies were observed, showed the highest antibacterial activity. On the other hand, QA8:TEG had the weakest antibacterial action. In the case of *E. coli*, QAm:TEGs from C8 to C14, on whose surfaces no bacteria colonies were observed, showed the highest antibacterial activity. On the other hand, QA16:TEG had the weakest antibacterial action [32]. However, the physicomechanical analysis showed that none of the QAm:TEGs had sufficiently high flexural properties or hardness. Their water sorption and residual monomer leachability were also excessively high [33].

Due to the detected intense antibacterial activity of QAm:TEGs, another series of copolymers was developed. They consisted of 40 wt.% Bis-GMA, 40 wt.% QAUDMA-m, and 20 wt.% TEGDMA (BG:QAm:TEG), analogous to the composition of another standard DCRM matrix, consisting of 40 wt.% Bis-GMA, 40 wt.% UDMA and 20 wt.% TEGDMA (BG:UD:TEG). They were characterized in terms of physical properties [34]. As can be seen from Table 1, they had low polymerization shrinkage, high glass transition temperature, and hydrophilic surfaces—except for BG:QA16:TEG and BG:QA18:TEG, which were hydrophobic. They also had sufficiently low water sorption and solubility, except BG:QA8:TEG and BG:QA10:TEG. These latter absorbed greater water quantity than is specified in ISO 4049 standard (40 µg/mm^3^) [35].

This work aimed to further characterize BG:QAm:TEGs for cytotoxicity on the L929 mouse fibroblast cell line and antimicrobial activity against *C. albicans*, *S. aureus*, and *E. coli*. The null hypothesis was that the BG:QAm:TEGs have antimicrobial activity and do not have cytotoxic effects on cells chosen for testing. Bioactive DCRM matrices, such as our proposed BG:QAm:TEGs, are highly desirable because infections caused by oral microflora are observed among adults and children [7,8]. These include tooth decay caused by bacteria and candidiasis caused by fungi. The available dental materials have an insufficient biocidal effect, and, in addition, growing antibiotic resistance is also a problem. This article may have cognitive and utility dimensions for developing potential novel DCRM systems with antimicrobial activity.

## 2. Materials and Methods

### 2.1. Monomer Synthesis and Crosslinking Copolymerization

The QAUDMA-m monomers were synthesized using the previously described procedure [31]. First, N,N-(2-hydroxyethyl)methylaminoethyl methacrylate (HAMA) was obtained using methyl methacrylate (MMA; Acros Organics, Geel, Belgium) and 2,2′-methyliminodiethanol (MDEA; Acros Organics, Geel, Belgium). Next, it was N-alkylated with alkyl 1-bromooctane,1-bromodecane, 1-bromododecane, 1-bromotetradecane, 1-bromohexadecane, and 1-bromooctadecane (Acros Organics, Geel, Belgium) to obtain 2-(methacryloyloxy)ethyl-2-hydroxyethylmethylalkylammonium bromides (QAHAMAs). Finally, the addition reaction of QAHAMAs to 1,6-diisocyanato-2,4,4-trimethylhexylene (TMDI; Tokyo Chemical Industry, Tokyo, Japan) was performed, and the QAUDMA-m monomers were obtained.

Next, 40 wt.% Bis-GMA (Sigma-Aldrich, St. Louis, MO, USA), 40 wt.% QAUDMA-m, and 20 wt.% TEGDMA (Sigma-Aldrich, St. Louis, MO, USA) were stirred at 60 °C to obtain homogenous compositions. As 0.4 wt.% camphorquinone (CQ; Sigma-Aldrich, St. Louis, MO, USA) and 1 wt.% 2-dimethylaminoethyl methacrylate (DMAEMA; Sigma-Aldrich, St. Louis, MO, USA), respectively the photoinitiator and accelerator, were added, the compositions were subjected to irradiation by utilizing a UV-Vis lamp (Ultra Vitalux 300, Osram, Munich, Germany) with a wavelength range of 280 to 780 nm for 60 min at room temperature (bulk polymerization). The PET film covering the photopolymerizing system prevented oxidation inhibition. Disc-like casts (diameter × thickness = 10 mm × 3 mm) were obtained. Once polished with sandpaper, they were used for the following tests: cytotoxicity, fungal adhesion, and fungal growth inhibition zone. The fungicidal and bactericidal tests employed powdered copolymers with a grain size of less than 25 µm. They were used to prepare suspensions in culture media.

**Figure 2:**
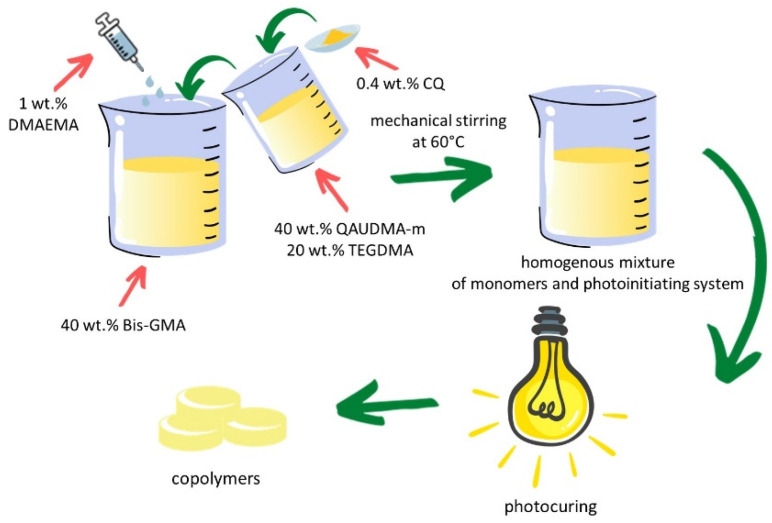
Monomer composition preparation and subsequent crosslinking copolymerization.

The names of copolymer samples were abbreviated as BG:QAm:TEGs (m—number of carbon atoms in N-alkyl chain in QAUDMA-m equal to 8, 10, 12, 14, 16, or 18). The copolymer of 40 wt.% Bis-GMA, 40 wt.% urethane-dimethacrylate (UDMA; Sigma-Aldrich, St. Louis, MO, USA), and 20 wt.% TEGDMA (BG:UD:TEG) was prepared as a reference (Figure 2).

### 2.2. Cytotoxicity

Cytotoxicity was determined according to the ISO 10993-5 standard [36] using the L929 mouse fibroblast cell line (ATCC-CCL-1). The L929 cell line was obtained from ATCC-CCL-1, NCTC clone 929 Areolar Fibroblast Mouse, delivered by LGC Standards Sp. z o.o. ul. Ogrodowa 27/29, Kiełpin 05-092 Łomianki.

Copolymer specimens of disc-like casts of 10 mm × 3 mm (diameter × thickness) were sanded clean and immersed in a culture medium (1 mL per 3 cm^2^ of the specimen surface) for 72 h at 37 °C with shaking at 80 rpm. The achieved extracts were mixed with fresh culture medium in a volume ratio of 1:1 and placed in a 96-well culture plate. Then, cell suspensions in a culture medium containing 5000 L929 mouse fibroblast cells were added to each well in the culture plate. Samples were incubated at 5% CO_2_ at 37 °C for 24 h (IncuSafe CO_2_ Incubator MCO-170AICUVD-PE PHCbi, Etten-Leur, The Netherlands). Next, 10 µL of Alamar Blue (G-Biosciences, St Louis, MO, USA) was added to each well, and samples were incubated at 37 °C for 4 h. Finally, fluorescence of aliquots was measured using a Varioskan LUX Multimode microplate reader (Thermo Scientific, Waltham, MA, USA).

### 2.3. Microbiological Properties

Microbiological properties were determined against a *C. albicans* (ATCC 2091) reference fungus strain as well as *S. aureus* (ATCC 25923) and *E. coli* (ATCC 25922) reference bacterium strains. Before testing, fungal and bacterial strains were cultured at 37 °C for 18 h (incubator POL-EKO, Wodzisław Śląski, Poland) in SDA (sabouraud dextrose lab-agar, Biomaxima, Lublin, Poland) and TSB (tryptic soy broth, Biomaxima, Lublin, Poland) medium, respectively.

#### 2.3.1. Fungal Adhesion

The adhesion of fungi to copolymer surfaces was determined on specimens in the form of disc-like casts of 10 mm × 3 mm (diameter × thickness). Specimen surfaces were finished with sandpaper before experiments.

Specimens were submerged in 1 mL of fungal suspension in a culture medium (~5 × 10^6^ CFU/mL) and incubated at 37 °C for 18 h. Next, the fungal suspension was removed with a Pasteur pipette, and samples were washed gently with 1 mL of sterile water. Washed specimens were placed in clean centrifuge tubes, soaked in 1 mL of sterile water, and vortexed (1 min, 3000 rpm) to remove fungi from sample surfaces. Next, 100 µL of the obtained fungal suspensions were diluted with 0.9% NaCl to achieve concentrations of 0.1, 0.01, 0.001, 0.0001, 0.00001, and 0.000001%. Then 100 µL of each solution was applied on agar plates (Sabourand agar, Diag-Med, Warszawa, Poland) and incubated at 37 °C for 18 h. Finally, the number of fungi colonies on plates illuminated by transmitted light was manually counted.

#### 2.3.2. Fungal Growth Inhibition Zone

The fungal growth inhibition zone diameter was determined for extracts obtained by soaking disc-like copolymer casts of 10 mm × 3 mm (diameter × thickness) in sterile water for seven days at room temperature.

We applied 100 µL of the fungal suspension in a culture medium (~5 × 10^8^ CFU/mL) to the agar plates. Next, 5 mm diameter holes were cut out. They were filled with 100 µL of the previously obtained copolymer extracts. Agar plates were incubated at 37 °C for 24 h. The inhibition zone diameter was measured.

#### 2.3.3. Fungicidal Activity

Copolymer powders of a grain with a diameter of less than 25 µm were used to prepare 25 mg/mL suspensions in SDA. Next, 20 µL of *C. albicans* fungal suspension in culture medium (~5 × 10^8^ CFU/mL) were introduced into the obtained copolymer suspensions, vortexed (10 s, 2000 rpm), and incubated at 37 °C for 18 h. Suspensions were vortexed again (10 s, 2000 rpm), and 100 µL was applied to agar plates. The agar plates were incubated at 37 °C for 18 h. The amount of fungal colonies was qualitatively assessed by visual comparison to the control.

#### 2.3.4. Bactericidal Activity

Copolymer powders of a grain with a diameter of less than 25 µm were used to prepare 25 mg/mL suspensions in TSB. Next, 20 µL of *S. aureus* and *E. coli* bacterial suspensions in a culture medium (~5 × 10^8^ CFU/mL) was introduced into the achieved copolymer suspensions, vortexed (10 s, 2000 rpm), and incubated at 37 °C for 18 h. Suspensions were vortexed again (10 s, 2000 rpm), and 100 µL was applied to agar plates (Müller-Hinton agar, Diag-Med, Warszawa, Poland). The agar plates were incubated at 37 °C for 18 h. The amount of bacterial colonies was qualitatively assessed by visual comparison to the control.

### 2.4. Statistical Analysis

Statistica 13.1 (TIBCO Software Inc., Palo Alto, CA, USA) software was used to perform statistical analysis. The experimental results represent the mean value of five measurements, accompanied by a standard deviation (SD). The statistical significance of the results was assessed by the non-parametric Wilcoxon test (level of significance (*p*) of 0.05).

## 3. Results

In this work, a set of dimethacrylate BG:QAm:TEGs were obtained by photopolymerization and characterized for cytotoxicity and microbiological properties. The BG:UD:TEG was used for comparison purposes.

### 3.1. Cytotoxicity

**Figure 3:**
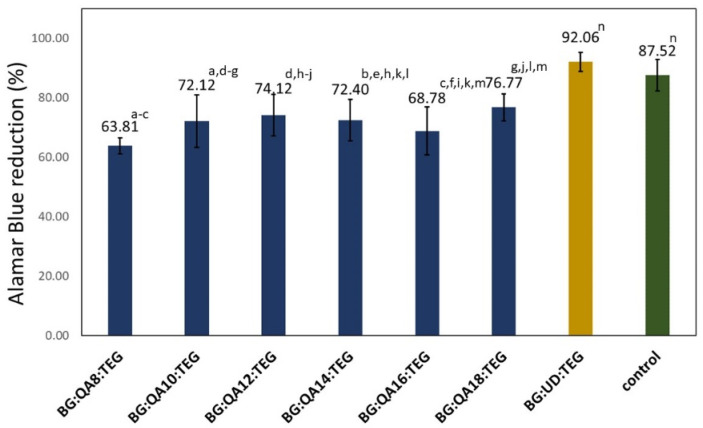
Viability of L929 mouse fibroblast cells after 24 h of incubation. Lowercase letters indicate statistically insignificant differences (*p* > 0.05) (non-parametric Wilcoxon test).

Figure 3 shows the results obtained for the Alamar Blue percent reduction. They correspond to the viability of the L929 cell line.

The cell viability determined for BG:QAm:TEGs was from 63.81 to 76.77%. Alamar Blue percent reduction values were statistically similar (*p* > 0.05), except for BG:QA8:TEG. The cell viability of the latter was lower (*p* ≤ 0.05) than that of BG:QA12:TEG and BG:QA18:TEG. Compared to the BG:UD:TEG, cell viability was reduced in all BG:QAm:TEGs (*p* ≤ 0.05).

**Figure 4:**
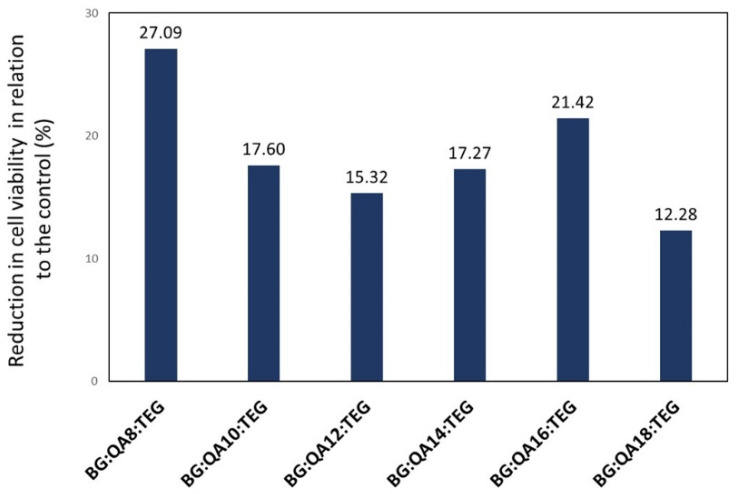
Reduction in cell viability of BG:QAm:TEGs relative to the control.

The viability of L929 mouse fibroblast cells in BG:QAm:TEGs was related to the control. The results of these calculations are shown in Figure 4. As can be seen, the reduction in cell viability observed for BG:QAm:TEGs ranged from 12.28 to 27.09%.

### 3.2. Microbiological Properties

**Figure 5:**
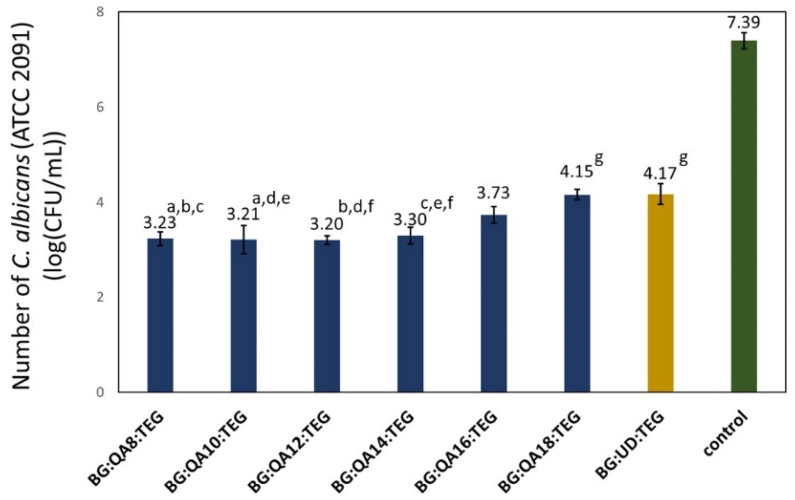
The results from the fungal *C. albicans* (ATCC 2091) adhesion test. Lowercase letters indicate statistically insignificant differences (*p* > 0.05) (non-parametric Wilcoxon test).

In Figure 5, the results of the fungal *C. albicans* adhesion test are shown.

The number of *C. albicans* colonies observed on BG:QAm:TEGs’ surfaces were from 3.20 to 4.15 log(CFU/mL). The number of fungal colonies on BG:QAm:TEGs with *Cm* from C8 to C14 was statistically similar (*p* > 0.05). The further increase in *Cm* up to C18 caused a significant increase in the log(CFU/mL) value (*p* ≤ 0.05). Compared to the BG:UD:TEG, the number of fungal colonies on almost all BG:QAm:TEGs was lower (*p* ≤ 0.05), except the BG:QA18:TEG. Its log(CFU/mL) value was similar (*p* > 0.05) to that determined for BG:UD:TEG.

**Figure 6:**
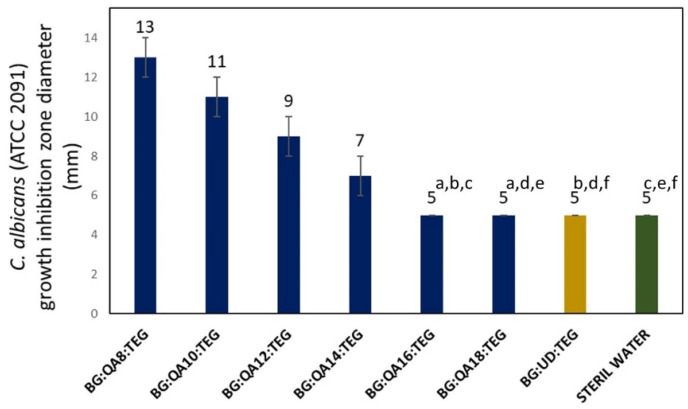
The fungal *C. albicans* (ATCC 2091) growth inhibition zone diameter. Lowercase letters indicate statistically insignificant differences (*p* > 0.05) (non-parametric Wilcoxon test).

In Figure 6, the results for the fungal growth inhibition zone are shown.

The fungal growth inhibition zones were only observed for BG:QAm:TEGs with *Cm* lower than C16. Their diameters ranged from 13 to 7 mm and decreased as the *Cm* increased. BG:QA16:TEG and BG:QA18:TEG showed no inhibition zone, similar (*p* > 0.05) to the BG:UD:TEG.

**Figure 7:**
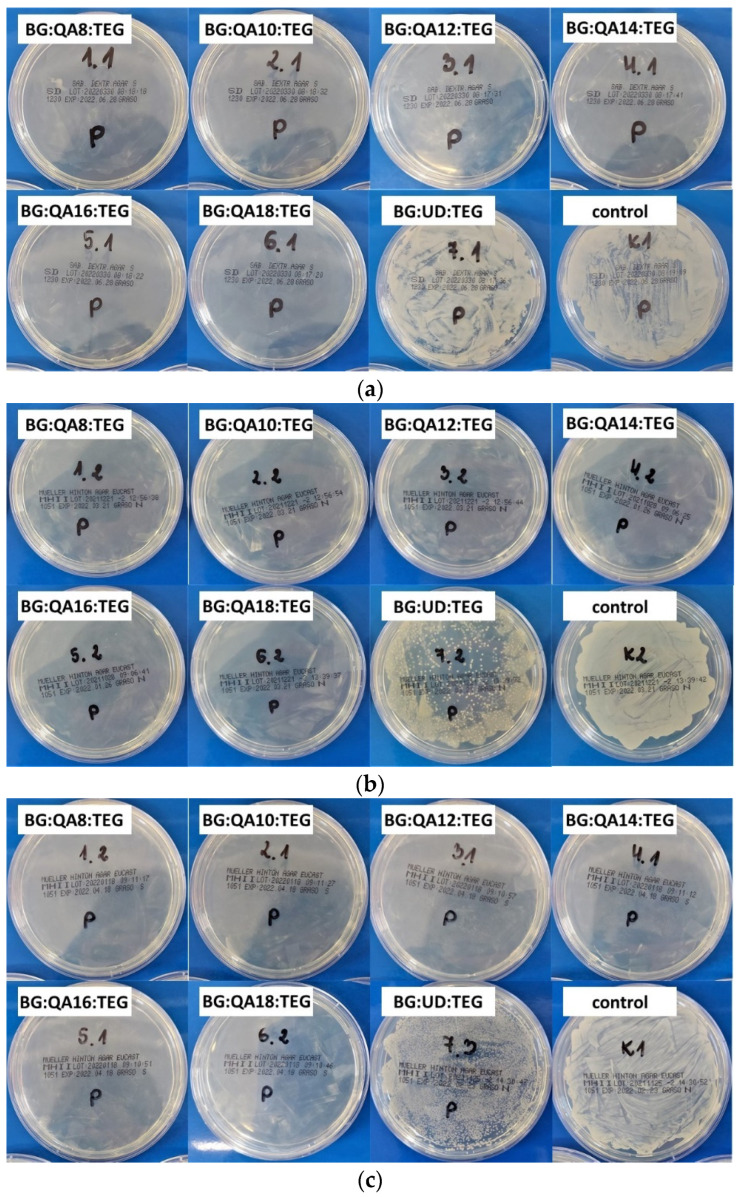
Results of microbial activity tests: (**a**) *C. albicans* (ATCC 2091), (**b**) *S. aureus* (ATCC 25923), and (**c**) *E. coli* (ATCC 25922).

In Figure 7, the results from the microbial activity test are shown.

Antimicrobial activity tests were performed by qualitatively assessing the number of microbial colonies on agar plates overlaid with 25 mg/mL copolymer suspensions in appropriate culture media, SDA, and TSB, respectively, for fungi and bacteria. As can be seen from Figure 7, no colonies of tested microbial strains (*C. albicans*—Figure 7a, *S. aureus*—Figure 7b, *E. coli*—Figure 7c) were observed for all BG:QAm:TEGs. The number of fungal colonies on the agar plate with the BG:UD:TEG suspension was similar to that of fungal colonies observed on the agar plate without any copolymer (control). On the other hand, the number of colonies of both bacterial strains on the agar plates with the BG:UD:TEG suspension was noticeably lower than the number of bacterial colonies observed on the agar plate without any copolymer (control).

## 4. Discussion

A set of six QAUDMA-m monomers with two QA groups substituted with N-alkyl chains of various lengths (*Cm* = C8, C10, C12, C14, C16, and C18) (Figure 1) were synthesized to obtain novel copolymers for dental applications. The concept assumed the total replacement of UDMA with QAUDMA-m in a typical dental BG:UD:TEG dimethacrylate copolymer.

**Figure 8:**
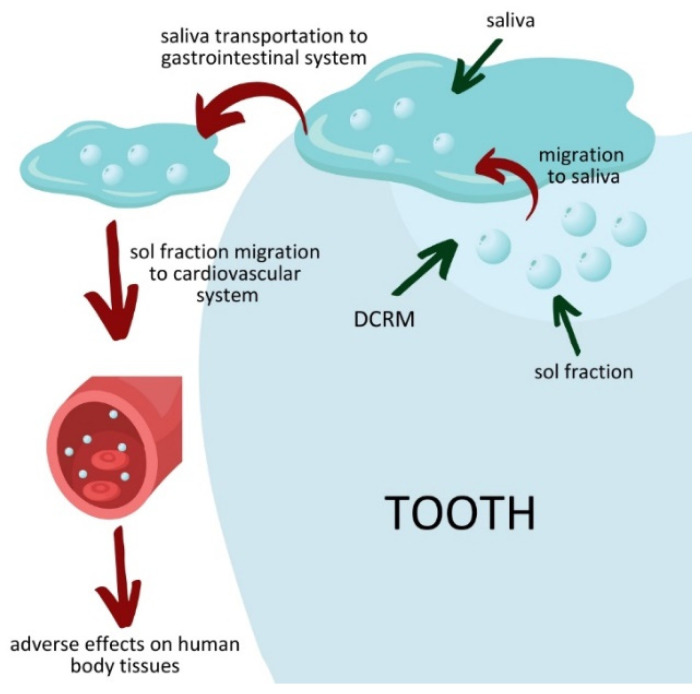
The origin of the DCRMs’ cytotoxic effect.

The cytotoxic effects of DCRM are closely related to sol fraction leaching [37,38,39]. The scale of this phenomenon depends on the combination of several factors, such as the matrix chemical structure, the degree of conversion [37,38], and oxygen inhibition on the DCRM surface [39]. The eluted substances can penetrate bodily tissues [38]. When they are cytotoxic, they cause inflammation, inhibition of cell division, or even necrosis (Figure 8) [40,41,42,43,44,45]. Therefore, the leachable fraction of dental material is standardized to 7.5 µg/mm^3^ [35].

The polymers with QA units have higher cytotoxicity than typical dental copolymers due to the presence of quaternary nitrogen atoms [46,47]. Therefore, analysis of their cytotoxicity is fundamental in dental materials studies.

This study evaluated the cytotoxicity of BG:QAm:TEGs compared to the BG:UD:TEG reference on the L929 mouse fibroblast cell line. In accordance with ISO 10993-5 standard [36], the results of cell viability allowed us to identify all tested BG:QAm:TEGs as noncytotoxic. This standard states that a reduction in cell viability by more than 30% to the control is considered a cytotoxic effect, whereas the decrease in cell viability of BG:QAm:TEGs was 15.32 to 27.09% (Figure 4).

**Figure 9:**
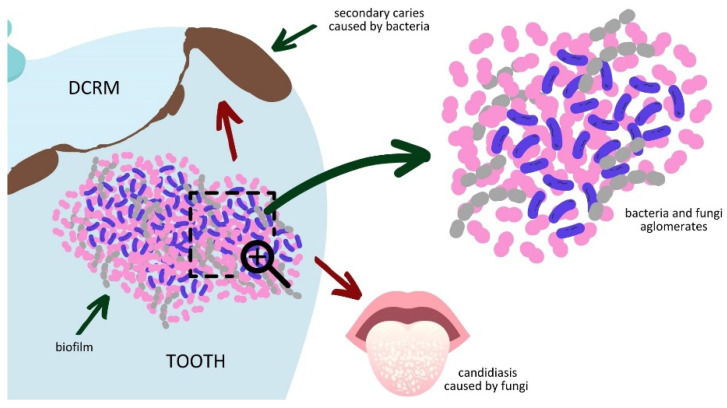
The action of bacteria and fungi comprising oral biofilm.

The biofilm formed on the tooth and dental filling surfaces consists of agglomerates of bacteria and fungi [48,49]. The acidic products of the bacterial metabolism cause enamel acidolysis and further secondary caries formation [50]. On the other hand, fungi cause candidiasis, considered the primary fungal infection of the oral cavity (Figure 9) [51]. The proliferation of bacteria and fungi is promoted by insufficient oral hygiene, primary and secondary caries, and untreated inflammation of the oral cavity [52,53]. All those factors indicate that the antifungal properties of dental materials are as crucial as antibacterial activity. Unfortunately, the antifungal activity of dimethacrylate copolymers containing QA groups has rarely been investigated.

In this study, the antifungal activity of BG:QAm:TEGs was examined against *C. albicans* (ATCC 2091) by evaluating the number of fungal colonies on the copolymer surfaces, inhibition zone diameters, and fungicidal effect of copolymer suspensions.

All BG:QAm:TEGs and BG:UD:TEG showed antifungal activity against *C. albicans*. The BG:QAm:TEGs with *Cm* lower than C16 showed similar and the highest antifungal activity. The number of fungus colonies found on their surfaces was 10^3^ orders of magnitude greater. Further increase in the *Cm* resulted in a significant increase in the log(CFU/mL) values. The BG:QA18:TEG had the highest log(CFU/mL) value, which simultaneously was almost the same as that for BG:UD:TEG.

**Figure 10:**
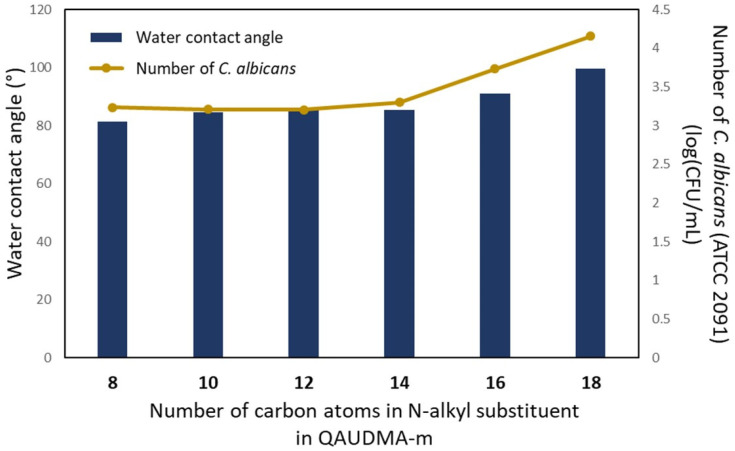
Comparing the water contact angle (WCA) values [28] with the number of fungi colonies on BG:QAm:TEG surfaces.

The literature shows that surface character is crucial in *Candida* biofilm formation [48]. Therefore, the results obtained for antifungal activity were related to those obtained for water contact angle (WCA) [34] (Figure 10). It can be seen that the greater the WCA values, the greater the number of fungus colonies on the BG:QAm:TEG surfaces. It can be concluded that the greater the surface hydrophobicity, the lower the antifungal activity of BG:QAm:TEGs.

Antifungal activity was also characterized by measuring inhibition zone diameter. The results showed that the greater the *Cm*, the smaller the inhibition zone diameter. However, the fungal growth-inhibitory effect was observed for BG:QAm:TEGs with *Cm* lower than C16. BG:QA16:TEG and BG:QA18:TEG did not show a fungal growth inhibition zone.

**Figure 11:**
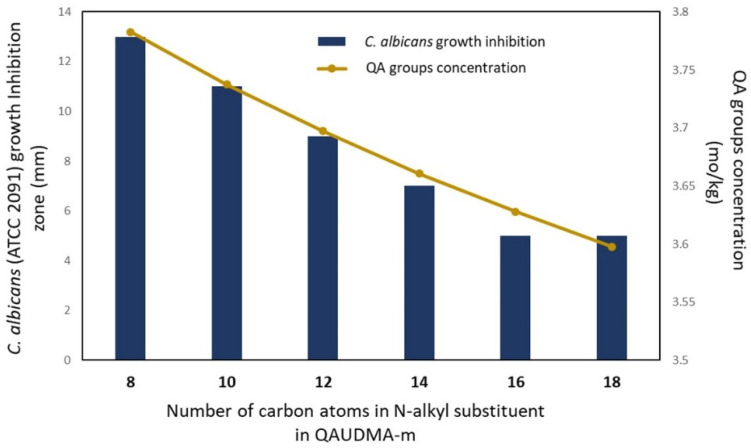
Comparing the *C. albicans* (ATCC 2091) growth inhibition zones with QA groups concentration (*x_QA_*) in BG:QAm:TEG compositions.

The results of the inhibition zone diameter were related to the concentration of QA groups (*x_QA_*) in the BG:QAm:TEG repeating units (Figure 11). As can be seen, the fungal growth inhibitory effects depend on the *x_QA_*. The lower its value, the lower the inhibition zone diameter. It can also be assumed that *x_QA_* of 3.63 mol/kg is the minimum below which no fungal growth inhibitory effect of the BG:QAm:TEG is observed.

The qualitative assessment of the amount of microbial colonies observed on agar plates overlaid with BG:QAm:TEGs suspensions indicated that the concentration of 25 mg/mL is sufficient to kill all tested microbial strains (*C. albicans*—Figure 7a, *S. aureus*—Figure 7b, *E. coli*—Figure 7c). On the other hand, the concentration of 25 mg/mL of BG:UD:TEG reference copolymer can be recognized only as sufficient to inhibit the growth of tested bacteria strains. An inhibitory effect was not observed for the *C. albicans* strain, as the visual assessment of the colony number was similar for BG:UD:TEG and the control. The results for BG:UD:TEG reference sample showed that this typical dental copolymer had antibacterial activity but not antifungal activity.

Data obtained for BG:QAm:TEGs suggests their antimicrobial activity and lack of cytotoxicity. However, a more comprehensive survey will be done to assess the possible therapeutic advantage of these copolymers. Microbiological characterization of BG:QAm:TEGs including the number of bacterial colonies on the sample surface and bacterial growth inhibition zone as well as mechanical properties will be the subject of another publication. Regarding cytotoxicity, as it was determined using only the L929 mouse fibroblast cell line, it should be checked using more cell lines, including human ones. Another area for development is that the results presented are limited to copolymers. If they were to be used as DCRMs’ matrices, testing on the model and commercial composites modified with QAUDMA-m should be carried out.

## 5. Conclusions

The modification of BG:UD:TEG by replacement 40 wt.% UDMA with the same amount of QAUDMA-m resulted in copolymers of low cytotoxicity and high antimicrobial activity against *C. albicans*, *S. aureus*, and *E. coli*.

BG:QAm:TEGs did not show cytotoxic effects on the L929 mouse fibroblast cell line, as the cell viability decreased by no more than 30% compared to the control. The Cm did not reveal the influence on cytotoxicity.

The antifungal effect against *C. albicans* was demonstrated by the reduction in the number of fungal colonies on copolymer surfaces, the occurrence of a growth inhibition zone, and the fungicidal effect of the 25 mg/mL copolymer suspensions. The antifungal effect depended on the *Cm* only from C14 to C18. It was revealed in correlations of a number of *C. albicans* colonies on copolymer surfaces with water contact angle (WCA) and fungal growth inhibition zone with a concentration of QA groups (*x_QA_*). The higher the WCA (lower surface hydrophilicity), the greater the number of *C. albicans* colonies on BG:QAm:TEG surfaces. The lower the *x_QA_*, the lower the inhibition zone diameter.

Antibacterial activity of BG:QAm:TEGs against *S. aureus* and *E. coli* was demonstrated by the bactericidal effect of the 25 mg/mL copolymer suspensions.

In summary, the null hypothesis has been verified, and BG:QAm:TEGs were found noncytotoxic and antimicrobial. However, further analysis should be done to demonstrate that they are safe bioactive biomaterials and do not pose a biological risk to patients.

## Figures and Tables

**Table 1:** Polymerization shrinkage (*S_e_*), glass transition temperature (*Tg_p_*), water contact angle (*WCA*), water sorption (*WS*), and solubility (*SL*) of BG:QAm:TEGs [34].

	*S_e_* (%)	*Tg_p_* (°C)	*WCA* (°)	*WS* (µg/mm^3^)	*SL* (µg/mm^3^)
BG:QA8:TEG	5.08	42.21	81.41	68.27	5.15
BG:QA10:TEG	5.48	45.81	84.68	48.42	5.18
BG:QA12:TEG	6.07	46.63	86.32	35.54	5.22
BG:QA14:TEG	6.14	47.83	85.52	34.43	5.58
BG:QA16:TEG	6.24	50.41	91.05	32.67	5.42
BG:QA18:TEG	6.40	50.81	99.53	25.94	5.54
BG:UD:TEG	8.35	55.90	80.76	11.71	1.12

## Data Availability

Data supporting the reported results are available from the authors upon reasonable request.
